# Hyaluronan, a Crucial Regulator of Inflammation

**DOI:** 10.3389/fimmu.2014.00101

**Published:** 2014-03-11

**Authors:** Aaron C. Petrey, Carol A. de la Motte

**Affiliations:** ^1^Department of Pathobiology, Lerner Research Institute, Cleveland Clinic Foundation, Cleveland, OH, USA

**Keywords:** hyaluronan, inflammation, glycobiology, Toll-like receptors, CD44, versican, TSG-6, inter-alpha trypsin inhibitor

## Abstract

Hyaluronan (HA), a major component of the extracellular matrix (ECM), plays a key role in regulating inflammation. Inflammation is associated with accumulation and turnover of HA polymers by multiple cell types. Increasingly through the years, HA has become recognized as an active participant in inflammatory, angiogenic, fibrotic, and cancer promoting processes. HA and its binding proteins regulate the expression of inflammatory genes, the recruitment of inflammatory cells, the release of inflammatory cytokines, and can attenuate the course of inflammation, providing protection against tissue damage. A growing body of evidence suggests the cell responses are HA molecular weight dependent. HA fragments generated by multiple mechanisms throughout the course of inflammatory pathologies, elicit cellular responses distinct from intact HA. This review focuses on the role of HA in the promotion and resolution of inflammation.

## Introduction

The association of increased HA deposition into the extracellular matrix (ECM) after tissue injury and during inflammatory disease has been recognized for over 25 years. Increased accumulation of HA has been demonstrated: in joint tissue of rheumatoid arthritis (RA) patients (1); in lung disease, both in humans (2) and animal experimental models (3–7); in inflammatory liver disease; during vascular disease (8, 9); in rejected kidney transplants (10) as well renal tissue of patients experiencing diabetic nephropathy (11); in the intestine of patients undergoing flares of inflammatory bowel disease (IBDs) (12), and mice with experimental colitis (13). Initially, HA was considered merely an inert space filling substance that had the capacity to surround itself with water molecules thus maintaining structure and preventing dehydration. The accompanying edema that occurs with HA deposition frequently impairs organ function.

Beyond the physical properties of a structural ECM molecule, however, it has become increasingly clear that HA provides cellular cues to regulate inflammation and tissue repair. The mystery is how and why does such a ubiquitous molecule produced in abundance by all vertebrates in every body tissue, foster inflammation under particular circumstances. This review focuses on discussing what is known regarding the mechanisms through which HA recruits and activates leukocytes in pathological inflammatory settings. Discussion will center on: (1) how HA is organized into a leukocyte recruiting matrix; (2) how it is degraded into fragments that are capable of signaling inflammatory responses via specific receptors; and (3) what are the known downstream effects and consequences of such activation. Over the past 15 years specific pathways signaled by HA have been defined and these have shaped the view of HA as a regulator of innate and acquired immunity, providing us with the concept that damaged ECM provides cues to surrounding cells to drive a protective response. In a dysregulated state, for example, the excess production of a pathological matrix, or the overproduction of HA fragments, HA is likely to contribute to chronic inflammatory conditions such as in RA, inflammatory bowels disease, atherosclerosis, and diabetes.

## Hyaluronan is a Size-Dependent Mediator of Inflammation

Data during the past two decades have established the fact that many of the cellular and biochemical processes mediated by HA are dependent upon the distribution of its molecular mass. HA polymers are comprised of repeating disaccharide units of glucuronic acid and *N*-acetyl glucosamine joined by alternating (β-1,3 and β-1,4) glycocidic linkages free of a protein core. An integral component of the ECM, HA polymers are traditionally considered as solvating, structural macromolecules largely responsible for supporting tissue integrity due to substantial viscoelastic properties (14). In normal tissues, HA exists as high-molecular-weight HA (HMW-HA) often with an average molecular weight of ~10^7^ Da that is capable of occupying a 1000-fold volume of water (15). HMW-HA is one of the principle components of the glycocalyx and is able to spatially exclude other molecules and cells, functioning as an anti-angiogenic factor. Several studies have shown that HMW-HA inhibits angiogenesis through reducing both the proliferation and migration of endothelial cells (7, 16, 17). At the extracellular surface HMW-HA interacts with surface receptors, prevents immune cell recognition, and blocks phagocytosis by macrophages (18, 19).

In cases of inflammation and tissue injury, HA is significantly more polydisperse and contains a variety of HA polymers with overlapping lengths and functions. Generally speaking, HA with an average molecular mass <500 kDa can be considered a fragment, although the molecular properties of such a size are quite different than those of a 50 kDa fragment. In most studies, the indicated sizes of HA represent the average molecular weight of a polydisperse distribution, and the preparations of HA used are not homogeneous with respect to size. As such, care must be taken when considering the effects of different polymer sizes.

Large HA polymers function as tissue integrity signals and serve to suppress the inflammatory response. As HMW-HA becomes depolymerized in inflammatory conditions such as RA, it loses its lubricant properties as noted in the synovial fluid of RA patients (1, 20). While it is unclear whether the generation of HA fragments is the result of HA catabolic enzymes, reactive oxygen species (ROS), a truncated product of the HA biosynthetic enzymes, or a combination of these mechanisms, it is clear that HA polymers of specific sizes contain distinct biological activities. HA exists as both a pro- and anti-inflammatory molecule *in vivo*, and these contradictory functions depend upon polymer length and which receptors the oligosaccharides engage. HMW-HA elicits protective anti-inflammatory effects that protect lung epithelial cells from apoptosis and is protective against liver injury, acting to reduce pro-inflammatory cytokines in a T-cell mediated injury model (3, 21). In regulatory T-cells, HMW-HA stimulates STAT5 signaling through CD44 crosslinking, promoting their maintenance and thereby inhibiting their proliferation. Conventional T-cell precursors stimulated with HMW-HA produce IL-10, and infusion of these cells attenuates the disease course in a murine model of colitis (22, 23).

**Figure 1:**
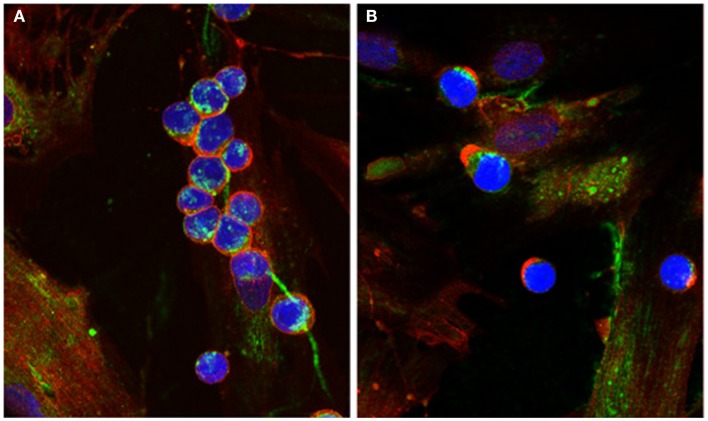
**Capping of CD44 on leukocytes**. **(A)** Monocytes bind to HA cables (green) produced by M-SMC in response to poly I:C. CD44 (red) is dispersed on the monocyte surface. **(B)** After 15 min incubation at 37°C, CD44 (red) is capped to one pole on the leukocytes, HA cables appear ragged (center panel) and HA is internalized. (Nuclei are blue).

The presence of cable-like structures comprised of HA, associated with binding proteins, has been noted in several inflammatory conditions including IBDs, atherosclerosis, diabetic nephropathy, and chronic kidney disease (24–29). HA polymers of indeterminate sizes originate from the surface of multiple cells and coalesce into large HA cables capable of spanning multiple cells and reaching several millimeters in length (30). The presence of HA cables at sites of tissue inflammation can still function as an anti-inflammatory polymer. Monocytes adhere tightly to the HA cables regardless of their activation state, and following adhesion the distribution of CD44 on the monocyte cell surface polarizes to form a “cap” while a portion of the HA cable is internalized (Figure 1). HA cables can function as a biological sink for monocytes and platelets, and these interactions likely modulate pro-inflammatory stimuli [see review in Ref. (31)]. The presence of HA cables is not unique to the inflammatory process, and cables are formed in response to ER stress, cycloheximide treatment, and viral infection (24, 32, 33).

HA catabolic hyaluronidase enzymes on the cell surface of platelets are capable of depolymerizing HA at neutral pH, and the subsequent fragments induce monocyte activation (34). HA fragments have also been shown to have a number of pro-inflammatory effects such as activation of macrophages and dendritic cells as well as stimulating transcription of inflammation-related genes including TNF-α, IL-12, IL-1β, and matrix metalloproteinases (35–37). However, it is important to note that some of the pro-inflammatory effects attributed to HA fragments likely result from contaminants in the HA preparations used. Caution should be taken with respect to the source and purity of HA for such studies. Polydisperse HA fragments with an average molecular weight of 200-kDa have been shown to stimulate chemokines, cytokines, growth factors, proteases, and nitric oxide by macrophages (38–44). In some cases, the signaling pathways engaged by HA fragments have been partially defined. Murine macrophages stimulated by HA fragments can activate the inhibitor of nuclear factor-κB pathway, which increases the expression of interleukin-1β and TNF-α (43). In smooth muscle cells, HA fragments promote cell-cycle progression by CD44-dependent activation of the Rac/ERK pathway, while the HMW-HA is inhibitory (45).

Although most of the work on low-molecular-weight HA fragments initially illustrated a pro-inflammatory response, a number of studies have shown that HA fragments can also be protective. In a DSS-induced murine model of colitis, intraperitoneal injection of polydisperse HA <750-kDa protects colonic epithelium in a Toll-like receptor (TLR) 4-dependent manner (46). Intermediate-molecular-weight HA <200 kDa stimulates the expression of human β-defensin 2 (HBD2) in human keratinocytes, through TLRs 2 and 4 (47). More recently, our laboratory has shown that HA-dependent induction of HBD2 in intestinal epithelial cells and in the colonic mucosa of mice is highly size specific. The strongest induction was observed with HA of 35-kDa average molecular weight, and was TLR 4 dependent (48).

## Hyaluronan-Binding Proteins and Their Roles in Inflammation

While many of the HA-binding proteins have been previously reviewed elsewhere (49), several HA-binding proteins are of particular importance with regard to inflammatory processes and will be discussed in the following section. The repeating nature of HA oligosaccharides and the specificity of protein–HA interactions likely dictate many of the mechanisms by which HA functions as either a pro- or anti-inflammatory molecule. The ECM, comprised predominantly of proteoglycans, glycoproteins, and collagens, is increasingly being viewed as having both structural and regulatory roles. Collectively, the ability of HA to function as either a pro- or anti-inflammatory molecule is dependent upon its size, microenvironment, localization, and availability of specific binding partners.

Inter-alpha-trypsin inhibitor (IαI) is one member in a family of serum proteoglycans secreted by the liver that function as serum protease inhibitors. It is reported to circulate in serum at concentrations between 150 and 500 μg/mL, comprises ~5% of total protease inhibitory activity of plasma, and elevated levels reported in inflammatory pathologies (50, 51). This proteoglycan is unusual as a single chondroitin sulfate chain is covalently attached to bikunin, a serine protease inhibitor, and another family of proteins termed heavy chain (HC) (HC1, HC2, HC3) are covalently linked to the chondroitin sulfate chain through an ester bond between C-terminal aspartate and the 6-hydroxyl of an *N-*acetylgalactosamine (52–54). In several inflammatory diseases, such as Crohn’s disease, asthma, osteo- and rheumatoid-arthritis (OA, RA), the increase in HA production is commensurate with increased levels of IαI heavy chains covalently attached to HA. In the case of Crohn’s disease, heavy chains of IαI are specifically associated with HA cable structures and are highly adhesive for naïve unactivated leukocytes, possibly in competition with inflammatory receptors (12, 55). Further, soluble HA containing HC1 and pentraxin 3 has been shown to induce apoptosis of activated, but not resting, neutrophils and macrophages (56). IαI and heavy chains are present at high levels in the synovial fluid of RA and OA patients, and HC complexes linked to HA have been shown to accumulate within inflamed tissues and correlates with disease severity (57, 58). Recently, the activity of TSG-6 in OA synovial fluid has been demonstrated as a biomarker for OA progression and the need for knee replacement (59). The transfer of HCs to HMW-HA is a reversible and dynamic event influenced by the availability of substrates, and smaller HA fragments (oligosaccharides) can act as irreversible HC acceptors and can disrupt highly adhesive properties of HC–HA (60). Studies of knockout mice lacking bikunin, which are unable to form IαI, have revealed an inhibitory role for IαI heavy chains in complement activation. Lack of IαI in mouse serum led to high levels of complement activation and was reversible upon injection of soluble IαI containing HCs (61). In humans, IαI does not appear to be a potent inhibitor of complement activation, affecting only early stages (62). In chronic inflammatory conditions such as RA, where IαI heavy chains and HA fragments accumulate to high levels and their effects may be more significant.

Tumor necrosis factor-stimulated gene 6 (TSG-6), which is capable of binding to both IαI and HA, catalyzes the transfer of HCs from the CS chain of IαI to HA by a *trans*-esterification reaction to form HC–HA, and has also been well studied in the context of ovulation (63, 64). The expression of TSG-6 is influenced by a number of factors including TNF-α, IL-1β, TGF-β, and LPS, in a highly cell type-dependent manner, and in many cases is coordinated with the synthesis of HA (65). While induced by inflammatory stimuli, TSG-6 has several protective features including enhancing the anti-plasmin activity of IαI and inhibition of MMPs and aggrecanases in cartilage (66, 67). Direct interaction between TSG-6 and the glycosaminoglycan (GAG) binding site of IL-8 has been shown to inhibit neutrophil migration by blocking the interaction of IL-8 with heparan sulfate on endothelial cell surfaces (68). The addition of TSG-6 to HA has been shown to enhance CD44-dependent lymphoid cell adhesion to HA in flow shear experiments (69). Interestingly, the Link module of TSG-6 alone can inhibit neutrophil migration and adhesion via exhibiting anti-inflammatory effects independent of HA and IαI (70). Recent studies have illustrated that: (1) the Link module of TSG-6 has binding sites for both HA and bikunin C4S; (2) TSG-6 is capable of binding non-covalently to HC1, HC2, and HC3 at affinities greater than with HA; and (3) although TSG-6 alone is capable of cross-linking HA into a dense, leukocyte-adhesive matrix, both the degree of condensation and adhesion are reversible by addition of IαI containing HC1 and HC2 (71). In a murine model of asthma, TSG-6 null mice are resistant to airway hyperresponsiveness and exhibit reduced levels of HA deposition, absence of HC–HA complexes and decreased eosinophilic inflammation (72). Some of these findings may be explained by the observation that airway smooth muscle cells stimulated with poly (I:C) and TSG-6 produce increased levels of cell-associated HA cables than treatment with poly (I:C) alone (73).

The adhesive properties of CD44+ cells to HA matrices depend upon the composition of proteins bound to HA. HC–HA complexes isolated from the synovial fluid of RA patients are highly adhesive for CD44+ leukocytes. These HA complexes likely contain several HA-binding proteins including HC3 and pentraxin-3, but were found to be free of TSG-6 (74). However, it has been recently shown that while TSG-6 enhances the interaction of HA and CD44, the *in vitro* addition of IαI, TSG-6, and HCs (HC1 and HC2) appears to counteract the enhanced binding of TSG-6–HA complex alone (69, 71). This is in conflict with prior reports, but suggests that HA-binding proteins themselves may regulate their own interaction with HA (71, 74). As suggested by Day and de la Motte, it is likely that the specific composition or organization of HA–protein complexes dictates the outcome of many cell–HA interactions (31).

Versican is a large CS-rich proteoglycan expressed at high levels by proliferating cells and mesenchymal cells and is deposited into the ECM during tissue remodeling and development. Versican is a member of the lecticans, structurally similar proteins that also include aggrecan, neurocan, and brevican. Versican consists of an amino-terminal globular domain (G1) and a carboxy-terminal globular domain (G3), separated by several CS attachment sites (GAG-α and GAG-β) between the two globular domains. The G1 domain consists of an immunoglobulin-like fold and a pair of link modules that bind five repeat disaccharides of HA with high affinity. This interaction is further stabilized by link protein itself, which binds both HA and versican (75, 76). Initial studies of versican showed anti-adhesive properties, which now appear to be mediated by the G1 domain (77–79). The G3 domain shares homology with the selectins, containing two epidermal growth factor (EGF) repeats, a C-type lectin domain, and a complement regulatory region. Four alternative splicing isoforms of versican (known as V0, V1, V2, and V3) result in truncation of the number of potential CS attachment sites (V1 and V2), with V3 lacking them. All isoforms retain the G1 and G3 domains and therefore the ability to bind to HA. The modular nature of versican can function as a highly diverse molecular constituent of the ECM capable of binding to a variety of factors involved in inflammatory processes.

Investigation into cancer growth and metastasis has implicated versican as having a central role driven by inflammatory stimuli. Versican, in either intact or fragmented forms containing the G3 domain, can enhance tumor cell migration, growth, and angiogenesis (80–82). In an *in vitro* screen for carcinoma-derived factors capable of activating macrophages, versican was identified as a potent enhancer of metastatic growth through TLR2 and co-receptors, TLR6 and CD14 (80). Versican has been suggested to contribute to HA fragment activation of macrophages, and enhanced cancer metastasis through induction of the hyaluronidases (83, 84). Several inflammation-associated cytokines, including transforming growth factor β1, 2, 3, and platelet-derived growth factor (PDGF), have been shown to increase biosynthetic levels of both versican and HA, while IL-1β, and IFN-γ have been shown to reduce levels of versican (85–91). Leukocyte trafficking and localization to regions of inflammation mediated by interaction with cell-adhesion receptors functions as a critical initiating step in the inflammatory cascade (92). Specific CS chains on versican preferentially bind to chemokines known to attract mononuclear leukocytes (93). Versican itself is capable of binding to a number of cell surface receptors present on leukocytes through interactions also mediated by CS chains, including both L- and P-selectins and CD44 (93–95). Direct binding of P-selectin glycoprotein ligand-1 (PSGL-1) by the G3 domain of versican has also been shown to cause aggregation of leukocytes (82).

Together, these HA-binding proteins contribute to the maintenance of tissue integrity and direct cell–ECM interactions in normal and pathological conditions. Many of the adhesive properties of HA polymers depend upon the presence of HA-binding proteins, and together with IαI, HCs, TSG-6, and versican contribute to a dynamic extracellular environment capable of directing cell adhesion and the production of inflammatory cytokines.

## Hyaluronan Catabolism and Generation of HA Fragments

Enzymatic degradation of HA is initiated by hyaluronidases (hyaluronoglucosaminidases, or HYALs), a family of endoglycosidases that hydrolyze the β-1,4 linkages between *N-*acetyl-hexosamines and glucuronic acid found in GAGs including some activity toward chondroitin 4- and 6-sulfates, and unsulfated chondroitin (96, 97). In humans, genes for six hyaluronidase family members have been identified to date: HYAL1–4, PH-20, and HYALP1. With the exception of the pseudogene HYALP1, each member encodes for protein products, and all but Hyal-3 have been shown to participate in either HA or CS catabolism [for a review see Ref. (98)]. While Hyal-3 does not appear to have a direct role in HA catabolism, it may be involved in an indirect fashion (99, 100). In somatic tissues, Hyal-1 and Hyal-2 function as the major hyaluronidases for HA degradation. In a rat model, exogenous addition of HA indicated that some turnover takes place locally at the site of intravenous injection, but the majority of clearance occurred in the lymph nodes and liver (101). However, other studies have suggested that local turnover is the major route of HA clearance and clearance through the lymphatic system is relatively minor when compared to local turnover by skin or skeletal muscle (102). These discrepancies may exist due to the size of HA used to measure local or lymphatic clearance, as it is likely that low-molecular-weight HA within tissue can be removed from the ECM by lymphatic drainage more easily than HMW–HA. Within cells, HA turnover occurs within the lysosomal compartment by concerted action of the endo-acting hyaluronidases, the lysosomal exoglycosidases, β-glucuronidase, and β-hexosaminidase, in a pH-sensitive manner (103, 104).

Of the six family members, Hyal-1 has been best studied. It is widely expressed and is found in plasma and urine as well as in several tissues including liver, kidney, spleen, and heart and cleaves HA to tetra- and probably also hexasaccharides (105, 106). Hyal1 is a 57-kDa glycoprotein that also occurs as a proteolytically processed 45-kDa form with two chains bound by a disulfide bond. The biological significance of two forms of Hyal-1 is not known, as both are present in tissues and cells while only the 57-kDa form is present in serum (107). Defects in HYAL1 were identified as the genetic lesion in the lysosomal storage disorder mucopolysaccharidosis IX (108). Loss of Hyal-1 activity results in accumulation of HA in serum as well as within the lysosomes of skin fibroblasts and macrophages (109). Once HA is within the lysosome, the lysosomal exoglycosidases appear to share some functional redundancy with Hyal-1 for HA turnover (97). Recombinant Hyal-1 has been reported to have a strict pH optimum near 3.7, with activity decreasing by ~75% ± 0.5 pH (107). However, several proteomics studies of lysosomes have not found Hyal-1 (or Hyal-2) in their analysis. This discrepancy may be due to the method of protein detection as most of these studies rely upon the fact that the majority of lysosomal hydrolases are targeted to the lysosome by the mannose 6-phosphate (M6P) pathway [for more details, see review in Ref. (110)], and therefore have utilized immobilized M6P-receptors as a purification method (111–120). Consequently, with the exception of one study (120), proteins trafficked to the lysosome by M6P-independent mechanisms are missing from these data sets. Other *in vitro* and *in vivo* studies have shown that Hyal-1, either in culture medium or serum, is taken up by endocytosis and does reach the endosomal–lysosomal network. This compartment, however, does not contain the classical late lysosomal hydrolases β-galactosidase or *N*-acetylglucosaminidase, and likely represents a late endosome (121, 122). These data fit well with the high levels of Hyal-1 observed in plasma and suggest that newly synthesized Hyal-1 reaches lysosomes through a secretion-recapture mechanism. The function of Hyal-1 within serum remains unclear.

Hyal-2 shares many of the features of Hyal-1: it is a 55-kDa glycoprotein widely distributed in a number of tissues, acid active, and found in two forms. Although it is frequently reported to be lysosomal, significant controversy exists around this point (123–125). Hyal-2 contains a glycosylphosphatidylinositol (GPI) linkage that tethers it to membrane surfaces where it can serve as the receptor for the jaagsiekte sheep retrovirus (84, 126, 127). GPI-anchored Hyal-2 has hyaluronidase activity when it is on the surface of human platelets (34). In certain cell types, a soluble, intracellular form lacking the GPI anchor has also been reported (128, 129). Initial studies of soluble Hyal-2 activity indicated that the enzyme is functional only in a narrow pH range around 3.7, similar to Hyal-1. Despite having a high degree of primary sequence homology to Hyal-1, Hyal-2 cleaves HMW–HA to generate 20-kDa fragments (approximately 50 disaccharides) *in vitro* (123, 125). A study of Hyal-2 expressed in HEK293 cells indicates strong membrane co-localization of Hyal-2 and CD44 with a pH optimum of 6.0 for membrane fractionated Hyal-2. Although some HA fragments generated by Hyal-2 were internalized, the majority was released into the medium (121). This is consistent with the finding that in breast cancer cells, Hyal-2 forms a complex with CD44 and the Na^+^–H^+^ exchanger 1 (NHE1), and HA degradation by Hyal-2 depends upon its interaction with CD44 at the membrane surface. However, controversy exists as to whether CD44 is present on the surface of platelets (130, 131). In culture, the interaction of CD44 and NHE1 leads to extrusion of H^+^ ions and a decrease in extracellular pH to 6.6 (132). Possibly, the distribution of Hyal-2 as either membrane-associated or soluble is cell-type or activation-state specific, and insertion into a lipid membrane can affect both the localization and the activity range of Hyal-2.

Degradation of HA by Hyal-1 primarily takes place within cells, and depends upon the ability of CD44 or other HA receptors to internalize HA fragments. Although significant levels of Hyal-1 are present in serum, to date it has not been shown to be functionally active in circulation. Interestingly, MPS IX patients deficient in Hyal-1 have been reported with plasma HA levels at 40 times normal (109). Thus it is unclear whether circulating Hyal-1 could potentially contribute to HA fragmentation. At this point in time, Hyal-1 appears to primarily contribute to intracellular HA turnover.

The ability of the hyaluronidases to depolymerize HA seems to be highly dependent on compartment and contribution of HA-binding proteins. The discrepancies in pH optimum reported in the literature may suggest that in a low pH environment, certain sizes of HA are easily degraded by Hyal-1 and Hyal-2 without the assistance of HA-binding proteins. It has been suggested that HMW-HA exists as a supramolecular structure capable of transitioning between secondary and tertiary structures in *in vitro* solutions (133, 134). However, other studies indicate that these interactions may not occur. Data demonstrating a lack of intrachain interactions open to competition, and an absence of amide-carboxylate hydrogen bonds required to form a twofold helical HA chain necessary for proposed models of secondary and tertiary HA structures, together suggest that intra-molecular structures are unlikely (135, 136). The structure of HA *in vivo* is likely to be dictated by the specificity, degree, and hierarchy of protein–HA interactions, and the ability of the hyaluronidases to degrade HA probably depends upon the conformation of HA chains. The finding of Hyal-2 in complex with CD44 at the plasma membrane is suggestive that HA-binding proteins can enhance the activity of HA degrading enzymes, and CD44 binding may provide Hyal-2 with a preferable conformation of HA. While Hyal-1 and Hyal-2 have been shown to prefer to cleave HA to tetrasaccharides and 50 disaccharides, respectively, these experiments have been performed *in vitro* for long periods of time and in most cases with exogenous HA free of HA-binding proteins. Given that neither enzyme has been shown to exhibit processivity, the sizes generated by Hyal-2 at the cell surface are more likely to be polydisperse and dependent upon the availability of HA-binding proteins.

While the hyaluronidases discussed above are at present the most plausible sources of HA fragments, they are not likely to be the only contributors. Recently, KIAA1199, a gene of unknown function involved in non-syndromic hearing loss and several forms of cancer has been identified as a HA-binding protein that contributes to HA degradation in cultured skin fibroblasts (137, 138). Interestingly, the authors show that siRNA-mediated knockdown of CD44 or Hyal-2 had no effect on HA degradation. Further, Hyal-1 did not appear to be expressed in Detroit 551 skin fibroblasts, and the majority of the HA depolymerization was lost upon KIAA1199 knockdown. However, it is important to note that although KIAA1199 appears to co-localize and co-precipitate with clathrin heavy chain, the authors were only able to detect depolymerized HA within media and were unable to detect fragments within the cell (137, 138). Future studies will determine whether KIAA1199 truly is a HA degrading enzyme.

Hyaluronan has long been suggested to protect articular tissues by absorbing ROS capable of degrading components of the ECM *in vitro* (139, 140). An additional mechanism suggested in the literature that could lead to HA fragmentation is the accumulation of ROS under conditions of injury or pathological inflammation. Accumulation of ROS in chronic inflammatory conditions has been noted, and direct depolymerization of HA by ROS has been illustrated largely *in vitro*, with some exceptions (141–146). In an epithelial airway culture system, xanthine/xanthine oxidase generation of ROS led to HA degradation and tissue kallikrein-mediated EGF receptor activation (147). However, ROS species themselves are capable of stimulating Hyal-2 gene expression via p38MAPK. In lentiviral-mediated Hyal-2 knockdown of cells exposed to ROS, HA depolymerization was not observed (148). Other species, including $O_{2}^{•-}$ and ^•^NO are present locally in inflammatory tissues and may recombine to produce peroxynitrite, which specifically degrades the HA, but not heparin/heparan sulfate (149–151). The contribution of ROS species to HA fragment generation will ultimately depend upon the relative ratio of these species, HA, anti-oxidants, and other substrates within the ECM. HA fragments generated directly by ROS species are functionally equivalent to enzymatic products is currently not known.

## Hyaluronan Signaling Receptors

CD44 is a type I transmembrane glycoprotein and is widely regarded as the major cell-surface HA binding protein (152). Widely studied in several contexts, CD44 interactions with HA have important roles in tumor metastasis, lymphocyte adhesion, T cell signaling, angiogenesis, and inflammation (153–156). CD44 contains a short cytoplasmic tail with multiple phosphorylation sites, a transmembrane domain, an extensively glycosylated variable region, and an amino-terminal HA binding domain containing a Link module (157–159). CD44 exhibits significant protein diversity partially due to variable splicing of exons, each encoding for a segment of the extracellular domain, and over a dozen isoforms have been discovered. Of the variable forms of CD44, the most common is termed hematopoietic or standard CD44 (CD44s, CD44H). The diversity of CD44 is further elaborated by the extent to which it is glycosylated. It contains up to two GAG attachment sites (CS or HS), and extensive *N-* and *O-*linked glycosylation alone can account for the majority of the molecular weight of CD44s. Many of the *N-*linked sites are found within the HA binding domain, and glycosylation of CD44 negatively regulates its ability to bind HA in some cell types (160). CD44 has been shown to require activation for high affinity HA binding, and one mechanism involves the enzymatic removal of terminal sialic acid from two *N-*linked glycans in the HA binding domain (161–163). Crosslinking of HA by binding proteins, such as TSG-6, can significantly alter the way in which HA is structured within the ECM and can alter the affinity of CD44 for HA (69, 158, 164).

CD44 is expressed on many cell types that contribute to inflammation including leukocytes, neutrophils, macrophages, chondrocytes, fibroblasts, epithelial, and endothelial cells. While the GAGs of CD44 can bind to cytokines, growth factors, and ECM proteins such as fibronectin, the majority of the functions of CD44 depend upon its ability to bind to HA (165). Just as the size of HA seems to dictate whether it functions as a pro- or anti-inflammatory molecule, studies have suggested CD44 shares contrasting roles. The molecular mechanisms dictating CD44s function may be driven by its affinity for HA. Several stimuli have been shown to activate or dampen the affinity of CD44 for HA. While if the transition from low- to high-affinity binding state (or vice versa) involves a conformational change has not been directly shown, it has been established that HA oligomers cause a ligand-induced conformational change and HA may have two mutually exclusive modes of binding to CD44 (159). Dimerization of CD44 may lead to activation of HA binding, and binding at the cell surface is the result of multiple weak HA–CD44 interactions, which are influenced by the size of the HA ligand (166–168). Interestingly, stability of HA binding appears to be size dependent and it is possible that stability itself may participate in signaling, which could explain the relationship between molecular weight and signaling (169). T cells are stimulated to bind HA by the cytokines IL-2 and TNF, as well as by chemokines such as MIP-1B, IL-8, and RANTES (170, 171). Monocytes can be stimulated to bind HA by a number of cytokines such as TNF-α, IL-1α, IL-1β, IL-3, IFN-γ, and LPS (163, 171–173). By contrast, some molecules including IL-4 and IL-13 inhibit CD44–HA binding (172, 174, 175). Antibodies to CD44 that interfere in HA binding attenuate inflammation in animal models of IBDs. In particular, CD44v7 has been strongly implicated in murine models of colitis as a potent inflammatory mediator. Disrupting CD44v7 isoforms with blocking antibodies induces apoptosis of mononuclear cells in both murine models of IBD as well as in monocytes isolated from inflamed mucosa of IBD patients (176–178). Mice deficient in all isoforms of CD44 develop normally, suggesting that other receptors can compensate for its loss (179). While CD44 is regarded as the primary cell-surface receptor for HA in many cell types, investigation into the mechanisms underlying HA fragment signaling has demonstrated that HA fragments are capable of signaling independently of CD44 (3, 48, 180, 181).

Toll-like receptors function as surveillance receptors, interacting with a number of microbial-derived molecules and activating the innate immune system in response to pathogen-associated molecular patterns (PAMPs). Increasingly, the TLRs are also shown to sense damage-associated molecular patterns (DAMPs) in response to injury as well (182). The idea that endogenous matrix degradation products act as regulators of cellular processes is not a new one, but with respect to GAG fragments, the role of HA is the best studied (183). HA is a component of the cellular coat on some pathogens including both groups A and C *streptococcus*, some strains of *Escherichia coli*, and *Pasteurella multocida*, and many of these pathogens also express hyaluronidases (184–186). The presence of a HA coat likely assists in evasion by the immune system, while the hyaluronidase enzymes may aid in colonization of the host. HA fragments functioning as DAMPs interact with TLRs, and thereby compete with HMW–HA to initiate receptor signaling. Studies of lysosomal storage disorders, where GAG fragments accumulate within the cell, have suggested that incomplete GAG degradation products are associated with increased activation of TLRs (187–189).

Ten TLR genes have been identified in humans to date, and both TLR4 – the primary signaling receptor for lipopolysaccharides, and TLR2 – a recognition receptor for mycoplasma and gram positive bacteria, are involved in recognition of fragmented HA (3, 35, 43, 48, 83, 190). While an increasing number of studies have shown that TLRs are involved in HA signaling, the underlying mechanisms remain unclear. In dendritic cells, data suggest that TLR4 is required for recognizing HA fragments of 4-, 6-, and 8-sugars, and recognition is independent of CD44, TLR2, or receptor for HA-mediated mobility (RHAMM) (36, 44). However, studies by Noble and colleagues have shown that macrophages isolated from either TLR2 or TLR4 knock out mice are still capable of chemokine gene expression induced by HA fragments, while macrophages from TLR2/4 double knockouts are not. Using a non-infectious bleomycin-induced lung injury model, they also demonstrated that TLR2/4 double knockout mice are protected from acute inflammation due to an impaired ability of macrophages to respond to HA fragments, but at the expense of epithelial cell repair (3).

The data presented by Noble’s group showed a requirement for MyD88, a downstream effector molecule shared by the TLRs, and for HA fragment signaling; HA-TLR signaling was abolished in MyD88-deficient macrophages (3). However, a recent study indicates that HA fragments of ~200 kDa are capable of inducing type I interferons by a TLR4 MyD88-independent pathway (191). These differences observed in signaling complex requirements may depend upon the size of HA used (135- vs. 200-kDa) or the spectrum of receptors present at the cell surface during the experiments. The specific receptor complexes and signaling pathways engaged appear to be highly cell type dependent. Taylor et al. have shown that although CD44, in some contexts, does not appear to be required for TLR4-mediated HA signaling, it can function in an enhancing role. The adapter molecules CD14 and MD-2 are required for TLR4 recognition of LPS, but CD14 is dispensable for HA signaling in cultured cells (35).

Despite a lack of biophysical evidence that TLRs bind directly to HA or other GAG fragments, interplay between these molecules as mediators of the inflammatory process is clear. Although data support CD44-independent TLR signaling by HA fragments in some cell types, it is not clear if there is competition between these receptors for HA fragment ligands or if they work in a co-operative fashion. Future studies are needed to define the signaling pathways activated by different sizes of HA fragments and the receptors utilized.

## Evidence for *in vivo* Importance of HA Fragments

*In vitro* studies have defined multiple signaling properties of HA fragments that occur in a cell type-specific manner and are mediated by a complicated arrangement of receptors. It is clear, however, that HA fragments possess important biological roles in innate immunity. A number of animal models have aided our understanding of how HA modulates inflammation in injury and disease. In a non-infectious lung injury model, HA fragments accumulate in lung tissue and require CD44^+^ macrophages for clearance before the tissue can be repaired (4). HA fragments are cleared as inflammation is resolved in wild-type mice, but fragments accumulate in CD44-deficient animals. The inability to remove HA fragments from the lung epithelium results in gross inflammation and impaired clearance of apoptotic neutrophils (4). At the cell surface, HMW–HA protects lung epithelium from injury through activation of NF-κB and suppression of apoptosis. Disruption of HA–TLR interactions results in loss of the protective effect of HMW–HA, and tissue injury is exacerbated (3).

HA-binding proteins have also been implicated in resolution of lung inflammation. IαI containing HCs expressed in the liver and normally secreted into serum, enter the lung through vascular leakage during inflammation. In bleomycin-challenged mice, IαI and HA normally appear to co-localize within inflamed lungs whereas in mice lacking IαI, increased levels of HA and cellular inflammation were noted. Together this suggests that HA–HC complexes may contribute to the resolution of lung injury (61, 192). Previous studies have shown HA–HC complexes are higher affinity for CD44 (74), and it is plausible that these HA-binding proteins work together to mediate tissue homeostasis within the lung. Some HA–HC preparations do not exhibit enhanced CD44 binding, and it is likely that the properties of HA–HC depend upon its exact composition of HA-binding proteins as well as the background of the cells involved (71). HMW–HA and CD44 interactions support cell survival, and in cases of injury, fragments accumulate and contribute to induction of inflammation. Clearance of HA fragments, the uptake of apoptotic neutrophils by macrophages, and restoration of HMW–HA pro-survival signals all depend upon CD44, and HA homeostasis is required to resolve lung inflammation and initiate tissue repair.

Though not discussed within the scope of this article, inflammatory mediators can promote angiogenesis, which facilitates chronic inflammation. New blood vessels can sustain a chronic inflammatory response by supplying a source of oxygen and nutrients to inflamed tissue and through transport of new inflammatory cells to the site of inflammation. HA polymers regulate the growth of new blood vessels through interactions with endothelial cells. HMW–HA is anti-angiogenic and inhibits endothelial cell proliferation, while fragmented HA is a pro-angiogenic stimulator. Endothelial cell interactions with HA are mediated in concert by CD44 and RHAMM (6, 7). Loss of CD44 disrupts angiogenesis *in vivo*, and this effect is due to the loss of CD44 expression in endothelial cells, as wild-type leukocytes or bone marrow-derived progenitor cells were unable to rescue the diminished angiogenic response. Interestingly, the endothelial cells deficient in CD44 are still capable of binding HA and exhibit normal migration, possibly due to compensation by RHAMM in these animals. In mice treated with an anti-CD44 antibody shown to block HA binding, neovascularization is drastically impaired, and new vessels cannot properly assemble endothelium-lined tubes (5). In tissues where new vessels are formed or become leaky due to injury, IαI from serum is readily available and can alter the adhesive properties of HA. In an *in vivo* matrigel angiogenesis model, mice lacking IαI exhibit drastically reduced vessel growth (192). However, it is worth noting that HA–HC complexes purified from the amniotic membrane appear to be anti-angiogenic, perhaps suggesting a different composition or organization (193). Endothelial cell interactions with a HA matrix depend upon the CD44–HA interactions that can be enhanced by IαI, and other HA-binding proteins, during vessel formation. Disruption of cell–matrix interactions leads to disrupted tube assembly and decreased stability of newly formed vessels.

Fragmentation of HA was recognized as one of the earliest biological markers for RA, and many animal models of RA are induced by ECM components such as type II collagen or aggrecan (14, 194, 195). Large amounts of HA containing HCs from IαI are found in the synovial fluid of RA patients, and HA–HC complexes contained within the synovium are highly adhesive for infiltrating leukocytes (74). Interestingly, mice incapable of forming HA–HC complexes, or treated with an anti-CD44 antibody, are more resistant to arthritis than wild-type littermates (196–198). By contrast, in a proteoglycan-induced arthritis model, mice deficient in CD44 only exhibit moderate to no resistance (199, 200). HA complexes containing TSG-6 and IαI accumulate to high levels at inflammatory sites and may support CD44-mediated leukocyte rolling and adhesion, while potentially having anti-inflammatory effects (69, 155, 201). High levels of TSG-6 have been reported in the secretory granules of mast cells within the inflamed joint tissues of mice, and intra-articular injection or cartilage-specific expression of TSG-6 have significant protective effects (66, 202, 203). It is possible that TSG-6 mediated crosslinking of HA results in a pro-adhesive but anti-inflammatory matrix for CD44+ cells (69). In the absence of CD44, the initial phases of leukocyte recruitment within inflamed synovial vessels are altered, and leukocytes appear to roll rather than adhere (204, 205). Leukocytes deficient in CD44 do not appear to interact as tightly with these HA complexes, suggesting that reduced adhesion contributes to the delayed onset of arthritis in these animals. Importantly, wild-type mice treated with anti-CD44 antibodies cross-link cell surface CD44, which rapidly disables leukocyte rolling. Antibody cross-linking of CD44 was followed by platelet deposition on the surface of granulocytes in the vascular beds of inflamed synovial tissue, and platelet-coated granulocytes were cleared from circulation leading to resolution of inflammation (205). These studies, in which different behaviors are observed when CD44 is either absent or blocked, are indicative of important differences reported in the literature with regards to the effects of HA fragments.

More extensive studies are needed to fully understand the specific molecular interactions between HA, HA-binding proteins, and HA receptors in the initiation and resolution of inflammation. The tissue microenvironment contributes significantly in regulating the inflammatory process. Defining the mechanisms by which inflammatory and protective HA fragments are generated, and how binding proteins influence their effects, will enable us to better understand the progression of inflammatory disease and perhaps reveal new therapeutic targets.

## Conflict of Interest Statement

The authors declare that the research was conducted in the absence of any commercial or financial relationships that could be construed as a potential conflict of interest.
